# Hounsfield Unit Utilization in Cervical Spine for Bone Quality Assessment: A Scoping Review

**DOI:** 10.3390/jcm14020442

**Published:** 2025-01-11

**Authors:** Riana Lo Bu, Rose Fluss, Yashraj Srivastava, Rafael De la Garza Ramos, Saikiran G. Murthy, Reza Yassari, Yaroslav Gelfand

**Affiliations:** 1 Department of Neurosurgery, Montefiore Medical Center, Bronx, NY 10461, USA; abbani@montefiore.org (R.F.); ygelfand@montefiore.org (Y.G.); 2 Dominick P. Purpura Department of Neuroscience, Albert Einstein College of Medicine, Bronx, NY 10461, USA

**Keywords:** Hounsfield units, cervical spine, computed tomography, clinical applications

## Abstract

Bone mineral density (BMD) is an essential indicator of bone strength and plays a crucial role in the clinical management of various spinal pathologies. Hounsfield units (HUs) calculated from computed tomography (CT) scans are a well-established, effective, and non-invasive method to determine bone density in the lumbar spine when juxtaposed to dual-energy X-ray absorptiometry (DEXA) scans, the gold standard for assessing trabecular bone density. Only recently have studies begun to investigate and establish HUs as a reliable and valid alternative for bone quality assessment in the cervical spine as well. In addition, multiple recent studies have identified cervical HUs as an accurate predictor of cage subsidence, an undesired complication of anterior cervical discectomy and fusion (ACDF) of anterior cervical corpectomy and fusion (ACCF) procedures. Subsidence involves migration of the spinal fusion cage into vertebral bodies, causing a loss of disk space, negatively altering spine alignment, and possibly necessitating further unwanted surgical intervention. Using the PRISMA-ScR checklist and the registered scoping review protocol (INPLASY2024100126), this review explores the current research on the use of cervical spine HU measurements as both a determinant of BMD and as a prognosticator of postoperative subsidence following cervical spine procedures (i.e., ACDFs and ACCFs) with the aim of improving clinical and surgical outcomes.

## 1. Introduction

Bone mineral density (BMD) is an essential indicator of bone strength and plays a crucial role in the clinical management of various spinal pathologies. Hounsfield units (HUs) calculated from computed tomography (CT) scans are a well-established, effective, and non-invasive method to determine bone density in the lumbar spine when juxtaposed to dual-energy X-ray absorptiometry (DEXA) scans, the gold standard for assessing trabecular bone density. Lumbar Hounsfield units are a well-validated, and a well-utilized alternative to DEXA as a determinant of bone mineral density. Until recently, it has been used as the main surrogate for DEXA during pre-operative assessment of bone density prior to both cervical and lumbar spine procedures. Only recently have studies begun to investigate and establish HUs as a reliable and valid alternative for bone quality assessment in the cervical spine as well. These studies are critical for accurate assessment of BMD and astute surgical planning in the cervical spine. A complication associated with the common procedures of anterior cervical discectomy and fusions (ACDFs), anterior cervical corpectomy and fusions (ACCFs), and cervical disk replacements (CDRs) is postoperative cage subsidence, which can negatively alter cervical spine alignment and possibly result in further unwanted surgical intervention. Bone quality is also critical for posterior instrumentation integrity and may influence the optimal construct length. The recent literature has investigated the reliability and utility of HUs obtained from CT scans as both a tool for calculating BMD and as an accurate prognosticator of cage subsidence. This scoping review aims to explore the existing literature on cervical Hounsfield units (HUs) and its predictive nature on bone density (summarized in Table 1), as well as cervical HUs as a predictor of subsidence after surgery for cervical spine pathologies (summarized in Table 2).

## 2. Materials and Methods

The PRISMA-ScR checklist, as outlined by Arksey and O’Malley (2005), was used to perform a scoping review with the registered scoping review protocol (INPLASY2024100126) (https://inplasy.com/inplasy-2024-10-0126/) (accessed on 29 October 2024). This review was performed by including all available studies (i.e., original research articles, reviews, and clinical guidelines published in peer-reviewed journals) within the scope of the review at the time of the literature search that examined computed tomography (CT) derived cervical Hounsfield units. A thorough literature search was performed in the PubMed, Web of Science, and Embase databases. The search strategy included the following keywords and MeSH terms: “Hounsfield units”, “cervical spine”, “computed tomography”, and “clinical applications.” The most up-to-date search was executed on 27 December 2024. Additional literature was identified by screening references of included studies. We considered studies that examined the HU measurement methods in the cervical region and adult populations with cervical spine pathologies. The excluded studies were non-English publications, duplicate studies, studies focused solely on non-cervical regions, case reports, cancer studies, radiotherapy studies, and any non-human studies.

The titles and abstracts of the resulting articles were screened by two independent reviewers (R.F. and R.L.). Full-text articles were retrieved for all relevant studies. The final selection was determined based on the eligibility criteria. Consultation or discussion with a third reviewer was used to resolve discrepancies.

A standardized extraction form was used to extract the following variables: authors, year of publication, study design, sample size, study country, patient demographics (age, gender, ethnicity, and disease group), CT scanner manufacturer, methods of HU measurement, key findings, and clinical applications. Both reviewers performed this process independently and consensus was reached on all the extracted data.

**Figure 1:**
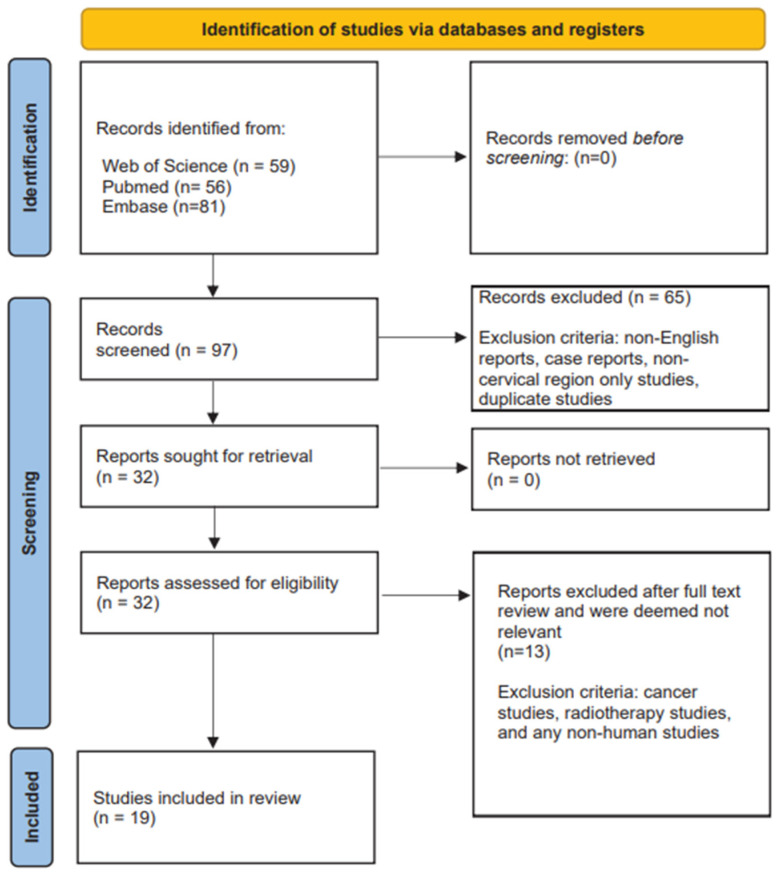
PRISMA flowchart of study selection.

The reviewers organized the data thematically by examining key areas such as the use of cervical HUs as a diagnostic for spinal conditions, as a prognosticator for poor clinical outcomes (i.e., subsidence), and methods for measuring cervical HUs. The reviewers performed a narrative synthesis to present their findings, with particular attention paid to gaps in the literature and directions for future studies. Although scoping reviews normally do not conduct formal quality assessments, we examined the study designs, methodologies, and limitations of the selected studies to provide context for the findings and assess the reliability and validity of HU measurements in clinical practice. A PRISMA flow diagram of the study selection process is represented in Figure 1.

## 3. Results

### 3.1. The Relation Between Cervical and Lumbar Hounsfield Units

Recent studies have shown a large discrepancy between the numerical range of cervical and lumbar Hounsfield units. Razzouk et al. examined the radiographic records of 165 patients without spinal neoplasm or infection and found a significant difference (*p* < 0.001) between the mean cervical and lumbar HUs (266.26 ± 88.69 vs. 166.45 ± 51.38, respectively) [2]. This discrepancy was confirmed by Simion et al., who assessed HUs from C3-L5 in 200 patients who underwent a CT scan. They found the mean spinal bone density was highest in the upper cervical spine (C3: 231.79 mg/cm^3^) and the lowest in the lumbar spine (L5: 155.13 mg/cm^3^), decreasing from cranial to caudal [1]. This reduction in HUs in the craniocaudal direction was confirmed by Schroder et al., who additionally found a significant difference between cervical and lumbar spine HUs [7].

Given the discrepancy in the range between cervical and lumbar Hounsfield units, Fluss et al. examined the correlation between the two and found that cervical and lumbar Hounsfield units had a moderately strong positive correlation (r = 0.79; *p* < 0.01) [5]. Razzouk et al. found a fair, positive correlation of 0.488 (*p* < 0.01) between the two [2].

### 3.2. Cervical Hounsfield Unit Measurement Methods

Five studies (three performed in Asia-China and Korea, one in the US, and one in Germany) have examined the variation in cervical HUs within the cervical spine levels. Liang et al. found a significant difference between the C3–C7 HUs, with the highest HU in the C4 vertebrae gradually decreasing HUs toward C3 and C7 [4]. Lin et al. confirmed that the mean HU was highest at C4 [3]. Lovecchio et al. found that each vertebral level had distinct bone mineral densities, and the lower cervical spine C6–T1 had the lowest HU [6]. In addition, the C7 bone mineral density was found to be significantly lower than those of the other cervical spine levels [20]. Simion et al. found that even within one vertebral level, specifically C2, the transitional area from the dens axis to the corpus had significantly lower HUs compared to other C2 regions [9]. Han et al. measured the mean HU of each of the C2–C7 vertebrae and compared the correlation of each vertebra with spine DEXA. They found fair to moderately strong correlations ranging from 0.52 to 0.65 in all the cervical segments, with the upper two cervical vertebrae (C2 and C3) having the highest correlation levels (r = 0.6–0.65) [7].

### 3.3. Correlation Between Cervical Hounsfield Units and Lumbar and Femoral T and Z Scores

To assess the reliability of using cervical Hounsfield units to determine bone mineral density, numerous studies have examined and found fair to moderately strong positive correlations between cervical HUs and DEXA. Colantonio et al. found a fair correlation between cervical HUs and femoral T-score (r = 0.531; *p* < 0.0001) [8]. These findings aligned with those of Han et al., who showed a moderately strong positive correlation of cervical HUs (C2–C3) with spine DEXA (r = 0.64–0.7) regardless of the degree of degeneration, patient age, or sex [7]. In addition, Lee et al. determined a moderately strong positive correlation between cervical HUs and DEXA in patients who had undergone both 1-level (0.57 < r < 0.71) and 2-level (0.59 < r < 0.66) ACDFs [11]. Fluss et al. examined the relation between average, lumbar, and femoral T- and Z-scores and cervical (C4-C6) HU values and found fair to poor correlations (0.436 > r > 0.274; all *p* < 0.01) [5].

### 3.4. Optimal Cervical Hounsfield Unit Cutoff for Determining Bone Density/Osteoporosis

To appropriately utilize cervical Hounsfield units as a corollary for DEXA, multiple studies have identified optimal cervical HU ranges and cutoffs for determining the various bone mineral density classifications (healthy, osteopenia, and osteoporosis). In a study examining patients in the Bronx, NY, an ethnically diverse population made up of predominantly Hispanic and Black patients, Fluss et al. examined the average cervical HU (C4–C6) for the various bone density classifications and determined 361.2 (95% CI 337.1–385.3) for healthy patients, 312.1 (95% CI 290.3–333.8) for osteopenic patients, and 288.4 (95% CI 262.6–314.3) for osteoporotic patients. There was a significant difference between the healthy and osteopenic patient cervical HUs (*p* = 0.0134), and those of the healthy and osteoporotic groups (*p* = 0.0304). They then determined 340.98 (73.5% specific, 57.9% sensitive, and AUROC: 0.665) as the threshold for assessing osteopenia and 326.5 (88.9% specific, 63.2% sensitive, and AUROC: 0.749) as the threshold for assessing osteoporosis [5].

A similar study performed by Han et al. in Korea found 284.0 ± 63.3 as the mean cervical HU (C3) for osteopenia, and 231.5 ± 52.8 as the mean HU for assessing osteoporosis (89.2% sensitive and 88.7% specific) [7]. A third study performed at the Walter Reed National Military found that their osteoporotic and osteopenic patients had significantly lower cervical HUs compared to the normal control patients (*p* < 0.0001). They determined the threshold of 447 HUs (sensitivity: 92%; negative predictive value: 82.1%) for low bone mineral density assessment (osteopenia and osteoporosis). They found no significant difference between male and female cervical HUs [8].

### 3.5. Cervical Hounsfield Unit as a Predictor of Subsidence

The cervical HU cutoffs for osteopenia and osteoporosis identified by the three studies discussed above differ by around 100 HU. This discrepancy raises questions about the underlying factors contributing to the varying cutoff values and the determination of an optimal threshold. Studies that have determined cervical HU cutoffs for predicting subsidence in ACDFs and ACCFs may provide insight into the debate of determining the optimal cervical HU for osteopenia/osteoporosis diagnosis and subsidence prevention.

Wang et al., a team at UCSF (University of California, San Francisco), examined the relation between cervical HU and subsidence rates after ACDFs. They found that the subsidence group had a significantly lower (*p* < 0.01, *t*-test) mean HU than the non-subsidence group (320.8 ± 23.9, n = 8 vs. 389.1 ± 53.7, n = 83). Using ROC (Receiver Operating Characteristic) analysis, they identified the HU threshold cutoff value of 343.7 (sensitivity 77.1% and specificity 87.5%) for predicting subsidence. They then performed a binary logistic regression analysis and confirmed lower preoperative HU to be a risk factor for postoperative subsidence [11]. Lee et al., a group in Korea, also divided their patients into two groups based on cage decrement and performed an ROC analysis to identify a HU threshold for predicting ACDF-related subsidence. They identified 530 as their cutoff HU value, and confirmed that a lower HU is an independent predictor of postoperative subsidence using multinomial regression analysis [13]. A third study, also performed in Korea, compared the mean HU of their high subsidence vs. low subsidence groups post ACDF and found the mean values of 284.1 for high subsidence and 316 for low subsidence [12]. Pu et al., a group in China, compared the HU value of their Zero-P subsidence and non-subsidence groups and found significant differences between age, axial and midsaggital, midcoronal, and midaxial HU between the two groups. Using ROC analysis, they identified the axial HU of 333.3 and midsaggital, midcoronal, and midaxial HUs of 326.8 as the optimal cutoff values for predicting Zero-P subsidence after ACDF [14].

Four studies, all performed in China, have examined the relation between cervical HUs and subsidence rates after ACCFs. Abudouaini et al. investigated the correlation between cervical HUs and titanium mesh cage subsidence after ACCF at around 18 months follow-up. They found a fair correlation between lower HUs (<330.5) and titanium mesh cage subsidence (r = 0.494) [15]. Ji et al. examined the risk factors for titanium mesh cage subsidence following ACCF at 24 months follow-up and found a global cervical HU value of less than 333 was significantly associated with subsidence [17]. Wang et al. also examined the effect of cervical HUs on early titanium mesh cage subsidence after ACCF with at least 12 months follow-up. They determined that a lower pre-op CT HU of less than 275 of the inferior vertebral body was an independent risk factor for early titanium mesh cage subsidence after ACCF [18]. Mei et al. examined the risk factors for early subsidence of 3D-printed artificial vertebral body (3D-PAVB) after ACCF surgery at around 1-year follow-up. Hounsfield units lower than 272 were found to be an independent risk factor for 3D-PAVB subsidence [19].

### 3.6. Limitations

During our literature review on the topic of the use of cervical Hounsfield units for bone quality assessment, we noted that despite there being a number of recent publications addressing the topic, there are currently no reviews summarizing and analyzing the current state of the field. Therefore, we decided to perform a scoping review to survey the current landscape of the use of cervical Hounsfield units as an alternative to DEXA. There are inherent limitations to this scoping review, which may affect the generalizability and reliability of our findings and conclusions. We searched a limited number of databases (Pubmed, Embase, and Web of Science) which may have limited our scope of findings. The studies described are widely heterogeneous in the demographics of their patient population (i.e., ethnicities, age, gender, and disease group), CT scanner manufacturer and scanning protocol, follow-up periods, sample size, measurement techniques, and results, which prevent us from being able to come to solid generalizable conclusions. To address these limitations and enhance the transparency and objectivity of our review, we have outlined the specifics of each study in Table 1 and Table 2. Despite these limitations, this scoping review does provide a summary of the current knowledge and state of the field, identifies knowledge gaps, and proposes possible future studies that may provide insights that advance the field and improve clinical and surgical outcomes.

## 4. Discussion

In summary, recent studies have shown that despite having vastly different numerical ranges, lumbar and cervical Hounsfield units positively correlate, measurement of cervical Hounsfield from any cervical vertebrae is a reliable corollary for DEXA regardless of the degree of degeneration, and lower cervical HUs is a risk factor for postoperative subsidence.

The large discrepancy between cervical and lumbar Hounsfield units demonstrates that the established lumbar HU cutoff values for determining osteoporosis (HU < 110–160) in the lumbar spine are inaccurate when applied to the cervical spine [21]. As lumbar Hounsfield units are around 100 HU lower than that of cervical Hounsfield units [1,2], this suggests that the lumbar HU cutoff for determining osteoporosis in the lumbar spine is too low to accurately capture most osteoporosis cases when applied to the cervical spine.

Despite having vastly different numerical ranges and, therefore, lack of translatability between lumbar and cervical HU cutoffs, the majority of studies have found fair to moderately strong positive correlations between cervical HU and DXA [2,3,7], with only one study finding a poor to fair correlation [5]. This may have been due to their inclusion of HU correlation with Z-scores in addition to T-scores. Z-scores compare patient bone density to those of similar age, sex, and body size, whereas T-scores compare patient bone density to that of a typically healthy 30-year-old. Because Z-scores include adjustments for age and are compared to a broader, more variable population, whereas T-scores are compared to a more uniform, optimal reference, correlations between Z-scores and DXA are often weaker than those of T-scores and DXA [22]. Overall, most studies have found fair to moderately strong positive correlations between cervical HU and DXA, supporting the reliability of using cervical HU as a corollary for bone mineral density assessment regardless of the degree of degeneration.

Surprisingly, most of the studies that have proposed cervical HU cutoffs for osteopenia and osteoporosis have identified widely disparate cutoff values ranging around 100 HU apart from each other despite using similar analyses [2,5,7]. This was also the case for the studies proposing HU cutoffs for subsidence. There was some overlap between the subsidence and osteopenia/osteoporosis cutoffs as the subsidence HU cutoff of 343.7 proposed by Wang et al. is close to the proposed HU cutoffs of 340.98 (osteopenia), 326.5 (osteoporosis) identified by Fluss et al. [5,11]. But there was still a large discrepancy between the remaining studies (summarized in Table 2), suggesting that there remains a large gap in the field in establishing a standardized cervical HU for determining poor bone density to prevent the development of subsidence.

This large discrepancy may be due to several diverse factors between the various studies, including different patient population demographics (i.e., ethnicity, age, gender), CT scanner manufacturer and scanning protocols, follow-up periods, sample size, and measurement techniques (i.e., the HU measurement method and the cervical vertebrae examined). The details for each study are outlined in Table 1 and Table 2.

It is well documented in the literature that racial and ethnic disparities affect bone health and outcomes [23]. Differences in socioeconomic factors, body composition, and diet are known to contribute heavily to racial/ethnic differences in bone mineral density [23]. For instance, Caucasians have been found to have slightly higher bone mineral density than Chinese American and South Asian populations and slightly lower bone mineral density than Black and Mexican populations [23]. Of the three studies cited in Table 1 that found cervical HU cutoffs for osteoporosis, one was performed in Korea and two were performed in the US (one in the Bronx, NY and one in Bethesda, Maryland, at the Walter Reed National Medical Center). The Korean study identified the mean HU of 284.0 +/- 63.3 and 231.5 +/- 52.8 for osteopenia and osteoporosis, respectively [7]. The study performed in the Bronx, NY, identified the thresholds of 340.98 for osteopenia and 326.5 for osteoporosis [5]. The study performed at the Walter Reed National Medical Center in Bethesda, Maryland, found a threshold of 447 HUs for assessing low bone mineral density [8]. The large variance of ~100 HUs between the three studies emphasizes the importance of considering ethnicity in future studies.

The various CT scanners used in the studies may have also contributed to the differing results between the studies. For instance, of the nine studies cited in Table 2, which determined cervical HU cutoffs to prevent subsidence and provided the specifics of their CT scanner manufacturer, none of them used the same CT scanner. It has been shown in the literature that two CT scanners from different manufacturers can produce different Hounsfield unit measurements for the same patient [24].

It is also possible that the discrepancy in Hounsfield units could be due to the studies measuring Hounsfield units from different cervical vertebrae to determine their averages and cutoffs. For instance, Fluss et al. took the average of C4–C6, Han et al. used the C3 value, and Colantonio et al. measured the C4 HU value [5,7,8]. As discussed earlier, cervical HU measurement from any cervical vertebrae should provide reliable information for assessing bone mineral density, as all the cervical spine segments have been found to have positive correlations ranging from 0.52 to 0.65 with spine DEXA [7]. However, the significant difference in HU value from C3–C7 may mean that the exact HU cutoff value determined by ROC analysis could differ based on which cervical vertebrae(s) were examined.

It is likely that the discrepancy in HU cutoff values is due to a combination of these factors. Therefore, to improve the accuracy and applicability of cervical HU thresholds, we propose that future studies standardize the specific cervical vertebral level examined and perform multicenter studies in diverse populations with the same CT scanner and protocols to determine cutoff values based on ethnicity, age, and sex. As all the studies presented here were retrospective, future prospective studies could help confirm the accuracy and reliability of the proposed thresholds. To increase the utilization of the cervical HU method, future studies should examine the profitability of this technique versus others, such as the MRI.

Therefore, it appears that the largest outstanding challenge in the cervical Hounsfield unit field is determining a standardized optimal cutoff for determining bone mineral density. Despite this challenge, the studies highlighted in this review show the exciting and important progress that has been made in establishing cervical HU values as a reliable and validated predictor of poor bone quality in surgical cervical spine patients. Our hope is for the use of cervical Hounsfield units to be integrated into routine preoperative assessments so that standardized cervical Hounsfield unit cutoffs in relation to age, sex, and ethnicity can become a standardized method for determining whether patients are optimal surgical candidates to help decrease the occurrence of subsidence and improve postoperative outcomes.

## 5. Conclusions

In this scoping review, we aimed to highlight the important integration of cervical HUs as measured from a cervical CT scan as a reliable measurement for BMD. Our review highlights the integration of HUs as a cost-effective approach to assessing bone mineral density. These findings further lend proof to the utilization of cervical HUs as a preoperative tool in predicting surgical outcomes. The evidence indicates a fair to moderately strong correlation between HUs and DEXA T-scores, highlighting the potential of HUs as an alternative or complementary measure for evaluating bone quality in the cervical region.

The importance of considering bone density in clinical decision-making and surgical planning is further highlighted in this review, as it correlates with the risk of cage subsidence following anterior cervical discectomy and fusion. Understanding the varying HUs across different cervical levels, combined with an individual understanding of preoperative HUs, may help correctly identify at-risk patients for subsidence. This review provides compelling evidence for the use of cervical HUs as a standard preoperative evaluation tool to reduce complications and improve patient outcomes. It also identifies and advocates for further studies addressing the current challenge in the field of establishing a standardized optimal cutoff for determining bone mineral density.

## Figures and Tables

**Table 1:** Cervical Hounsfield unit as a determinant of bone density.

Citation	Study Design / Limitations	Vertebrae Used for HU Measurement	Results / Conclusion
Simion et al., 2024 [1].	**Study design:** retrospective, single center **Objective:** to use Hounsfield units to determine trabecular cervical, thoracic, and lumbar spine bone density values **Sample size/follow-up:** *n* = 200; 2-year follow-up **CT scanner:** 256-slice Multi Detector CT Scanner GE Healthcare Revolution **Patient demographics:** 153 males and 47 females; average age 47.05 years old; ethnicities were not specified (the study was performed in Germany) **Inclusion/Exclusion criteria:** exclusion: fractures in more than three vertebrae, prior spine surgery, osteochondrosis in more than 3 vertebrae, prominent scoliosis or kyphosis, and implanted materials **Statistical significance of results**: significant difference found between cervical, thoracic, and lumbar spine bone density values (*p* < 0.001) **Limitations:** only used CT scans in the context of trauma; may have included patients with pre-existing conditions; did not collect data on medications that may impact bone density	C3–L5	Bone density values decreased from upper cervical spine to lower thoracic spine (C3: 231.79 mg/cm^3^; L5: 155.13 mg/cm^3^; *p* < 0.001)
Razzouk et al., 2024 [2].	**Study design:** retrospective, single center **Objective**: to evaluate the correlations between cervical, thoracic, and lumbar hounsfield units **Sample size:** *n* = 165; 1-year follow-up **CT scanner:** GE Discovery 750 HD 64 slice CT Scanner **Patient demographics:** 105 females and 60 males; 52 patients were caucasian (31.5%), 73 patients were Hispanic (44.2%), 21 patients were African American (12.7%), 11 patients were Asian (6.7%), and 8 patients were Native American or Pacific Islander (4.8%). **Inclusion/exclusion criteria:** exclusion: spinal neoplasm or infection, spinal instrumentation, and IV contrast-enhanced CT **Significance/Strength of correlations:** fair to moderately strong correlations of 0.574 (cervical HUs vs. thoracic HUs), 0.488 (cervical HUs and lumbar HUs), and 0.686 (thoracic HUs and lumbar HUs) **Limitations:** limited medical history exclusion; no stratification of patients based on bone density	C3–C7, T8–T12, and L1–L4	Mean cervical HUs (C3–C7): 266.26 ± 88.69; mean thoracic HU (T8–T12): 165.57 ± 55.06; and mean lumbar HUs (L1–L4): 166.45 ± 51.38 were all significantly different with positive correlations. HUs did not significantly differ based on sex, age, height, weight, or ethnicity.
Lin et al., 2022 [3].	**Study design:** retrospective, single center **Objective:** to assess the correlations between C1–T1 vertebrae **Sample size:** *n* = 71; 1-year follow-up **CT scanner:** Siemens Healthineers AG; Erlangen, Germany **Patient demographics:** mean age: 53.3 ± 12.7; 46% female and 54% male; ethnicity was not specified (study was performed in China) **Inclusion/exclusion criteria:** exclusion: previous spinal instrumentation, missing/incomplete pre-op CT imaging, missing C7 and T1 imaging, ankylosing spondylosis, inflammatory diseases, and tumor **Significance/Strength of correlations:** fair to very strong correlations between cervical spine levels (0.412 to 0.951) **Limitations:** possible lack of applicability to general population due to presumably predominantly Chinese population	C1–T1	Mean HUs from C1–T1 are all significantly correlated. Highest mean HUs were found in the mid-cervical spine (C4). Mean HUs were not correlated with age, sex, and BMI.
Liang et al., 2022 [4].	**Study design:** retrospective, single center **Objective:** to use HUs to assess bone mineral density distribution within the cervical vertebrae **Sample size:** *n* = 324; follow-up period not specified; 3 year span of data **CT scanner:** Siemens Sensation 64; Erlangen, Germany Patient demographics: mean age 53.3 ± 12.7; all Asian ethnicity **Inclusion/exclusion criteria:** Inclusion: degenerative cervical spine disease patients, aged between 21 and 80 years old; exclusion: posterior longitudinal ligament ossification, spondylolisthesis, spinal deformity, congenital spinal stenosis, tuberculosis, tumors, fractures, inflammation, coronal and sagittal imbalance, prior cervical surgery, and alcohol and tobacco addiction **Significance/Strength of correlations:** significant differences found between HUs of C3–C7 (*p* <0.01), and HUs from the upper and lower parts of the C6 and C7 vertebrae (*p* < 0.01) **Limitations:** lack of applicability to general population due to all Asian patient population	C3–C7	C3–C7 HUs were all significantly different, with the highest HUs in C4, decreasing toward C3 and C7. HUs decreased in association with Pfirrmann classification-based disk degeneration.
Fluss et al., 2024 [5].	**Study design:** retrospective, single center **Objective:** to determine the correlation between cervical HUs and DEXA T and Z scores, and to determine cervical HU thresholds for bone quality classification **Sample size:** *n* = 128; 2 year span of data **CT scanner:** General Electric Lightspeed VCT 64 Slice CT Scanner **Patient demographics:** average age and ethnicity not specified (study was performed in Bronx, NY) **Inclusion/exclusion criteria:** exclusion: prior spinal instrumentation or fracture in the region of interest **Significance/Strength of correlations:** fair to poor positive correlations (0.436 > r > 0.27) between cervical (C4-C6) HU values and average, lumbar, and femoral T- and Z-scores **Limitations/analysis:** limited medical history exclusion, poor to fair correlations found between cervical HUs and T- and Z-scores	C4–6; L1–4	Significant positive correlations were found between (1) cervical HUs and T- and Z-scores (average, lumbar, and femoral) (0.436 >; r > 0.274; all *p* < 0.01) and (2) cervical and lumbar HUs (r = 0.79; *p* < 0.01) Average cervical HUs were identified for: 1) healthy patients—361.2 (95% CI 337.1–385.3); 2) osteopenic patients—312.1 (95% CI 290.3–333.8); and (3) osteoporotic patients—288.4 (95% CI 262.6–314.3). Significant differences were found between healthy and osteopenic patient HUs, and healthy and osteoporotic patient HUs Identified thresholds of 340.98 (73.5% specific and 57.9% sensitive) for determining osteopenia; 326.5 (88.9% specific and 63.2% sensitive) for determining osteoporosis
Lovecchio et al., 2024 [6].	**Study design:** retrospective, single center **Objective**: to determine if cervical HUs vary by level from C2-T1 **Sample size:** *n* = 224; 4 year span of data **CT scanner:** 16-MDCT scanner (MX8000 Philips Healthcare, Andover, MA) **Patient demographics:** mean age 56.7 ± 12.7; 63.5% male and 36.5% female; 90.1% white, 2% Black, 1.5% asian, and 6.4% other **Inclusion/exclusion criteria**: inclusion: patients with degenerative spine pathology; exclusion: prior spinal instrumentation, pathophysiologic cervical spine conditions, osteoporosis, and outside cervical CTs **Significance/Strength of correlations**: significant differences in bone density between C6-T1 (*p* < 0.001) **Limitations:** lack of applicability to general population due to predominantly white patient population	C2–T1	The lowest HU values were found in the lower cervical spine (C6-T1)
Han et al., 2022 [7].	**Study design:** retrospective, single center **Objective:** to determine the most reliable method of measuring HUs for reflecting bone mineral density **Sample size:** *n* = 439; CT and DXA within 1 year of each other **CT scanner:** Siemens Medical Solutions; Erlangen, Germany **Patient demographics:** mean age: 55.8 ± 15.6 years; range: 19–94, male (55.8 ± 17.1 years) and female (56.4 ± 14.0 years); ethnicity was not specified, study was performed in Korea **Inclusion/exclusion criteria:** inclusion: patients ≥ 18 years old; *exclusion:* major spine trauma, tumor, congenital injury, prior spine instrumentation, and infection **Significance/Strength of correlations:** fair to moderately strong correlations found between C2–C7 HUs and DXA (0.52 to 0.65) **Limitations:** possible lack of applicability to the general population due to presumably predominantly Korean patient population	C2–C7	Cervical HUs (C2–C7) and lumbar and femoral T-scores are positively correlated (0.65 > r > 0.52). Identified mean HUs of 284.0 ± 63.3 and 231.5 ± 52.8 for osteopenia and osteoporosis. Spine DXA score correlated with HUs regardless of degeneration classification, sex, or patient age
Colantoio et al., 2020 [8].	**Study design:** retrospective, single center **Objective:** to determine the correlation between the C4 HUs and DXA T-score **Sample size:** *n* = 149 **CT scanner:** N/A **Patient demographics:** ethnicities not specified, study performed at Walter Reed National Medical Center **Inclusion/exclusion criteria:** inclusion: patients with cervical CT and femoral neck DXA scans **Significance/Strength of correlations:** significant differences in HUs (*p* < 0.0001) between the low BMD (osteopenic and osteoporotic) and normal group in study **Limitations:** only examined the relation between one cervical vertebral level and DXA	C4	Osteoporotic and osteopenic patient HU values differed significantly from normal patients. Identified threshold of 447 HUs for assessing low BMD (92% sensitive, and 82.1% negative predictive value)
Simion et al., 2023 [9].	**Study design**: retrospective, single center **Objective**: to evaluate trabecular bone density of C2 using HUs **Sample size:** *n* = 198; 2-year period **CT scanner:** 256-slice Multi Detector Ct Scanner GE Healthcare Revolution **Patient demographics:** ethnicities were not specified; the study was performed in Germany **Inclusion/exclusion criteria**: exclusion: spinal pathologies, prior spinal instrumentation, osteochondrosis, spondylodiscitis, and artifacts from implanted materials **Significance/Strength of correlations:** median bone density varied in the different C2 regions with the transition area from dens axis to corpus being significantly lower than the other C2 areas (*p* < 0.001); significant decrease in bone density after age 50 (*p* < 0.001) **Limitations**: only one cervical vertebral level was examined; possible lack of applicability to general population due to presumably predominantly German patient population	C2	Transitional area from dens axis to corpus had significantly lower bone density compared to other C2 regions Significant decrease in bone density identified after age of 50 years in both men and women
Schroder et al., 2022 [10].	**Study design**: cross-sectional observational study on human cadaver vertebrae **Objective**: to examine associations between biochemical resilience and cancellous bone with low bone density **Sample size:** *n* = 13 body donors **CT scanner:** GE Revolution EVO/64 slice CT/lateral scanogram **Patient demographics:** 4 men and 9 women; mean age: 84.3 ± 8.4 years, ethnicities were not specified; the study was performed in Germany **Inclusion/exclusion criteria**: inclusion: 22 vertebral bodies; *exclusion*: anatomical deformities, tumors, Paget’s disease, spinal fusion, prior spinal instrumentation involving foreign material, and growth retardation **Significance/Strength of correlations:** HUs were reduced in the craniocaudal direction, with cervical and lumbar spine HUs significantly differing (*p* < 0.001) **Limitations**: small sample size	C2–L5	HUs were reduced in the craniocaudal direction

**Table 2:** Cervical Hounsfield unit as a predictor of subsidence.

Citation	Study Design/Limitations	Vertebrae Used for HU Measurement	Results/ Conclusion
Wang et al., 2020 [11].	**Study design**: retrospective, single center **Objective:** to evaluate the association between HUs and subsidence rates after ACDF **Sample size:** *n* = 91; minimum of 1-year follow-up **CT scanner**: not specified **Patient demographics**: mean age: 54.5 ± 11.0; 54 females and 37 males; ethnicity was not specified; the study was performed in San Francisco, California **Inclusion/exclusion criteria**: inclusion: single-level ACDF for degenerative spinal conditions; *exclusion*: prior spinal instrumentation at studied level, trauma, infection, tumor, cervical kyphosis > 5%, and standalone integrated screw and cage devices **Significance/Strength of correlations**: moderately strong negative correlation between HUs and segmental height loss (r = −0.735) **Limitations**: could have included ethnicity in their assessment of variables affecting hounsfield units and subsidence	Vertebrae above and below graft placement	Mean HU value of 320.8 ± 23.9 (*n* = 8) for the subsidence group was significantly lower than the non-subsidence group (389.1 ± 53.7, *n* = 83) Determined negative correlation between HUs and segmental height loss (r = −0.735; *p* = 0.01) Identified HU threshold of 343.7 (sensitivity 77.1%, specificity 87.5%) for subsidence Lower HU pre-op is a risk factor of subsidence postoperatively.Subsidence rate/distance were not significantly different between allograft and polyetheretherketone (PEEK) materials
Lee et al., 2022 [12].	**Study design:** retrospective, single center **Objective**: to determine independent risk factors for postoperative subsidence **Sample size:** *n* = 40; minimum of 1-year follow-up **CT scanner:** not specified **Patient demographics:** mean age: 52 years; 19 male and 21 female **Inclusion/exclusion criteria:** inclusion: (1) neck pain and radiculopathy patients >3 months based on MRI; (2) cervical myelopathy involving acute rupture of the cervical disk; and (3) C7 and T1 vertebral body visible on pre-op cervical sagittal plane **Significance/Strength of correlations:** moderately strong positive correlation between lumbar BMD and cervical HUs (r = 0.733) **Limitations:** small sample size; DEXA not performed on all patients in the study	C2–7	Lumbar BMD values significantly correlated with cervical HUs values (r = 0.733; *p* < 0.001) Identified cervical HU cutoff of 530 for subsidence (AUC: 0.727, sensitivity: 94.7, specificity: 42.9). Determined HU value and intraoperative distraction as independent factors for postoperative subsidence
Lee et al., 2021 [13].	**Study design:** retrospective, single center **Objective**: to examine the correlation between HUs, DXA, and subsidence after both 1 and 2-level ACDF **Sample size**: *n* = 235; 4-month follow-up **CT scanner**: N/A **Patient demographics:** ethnicity was not specified; the study was performed in Korea **Inclusion/exclusion criteria:** inclusion: 1 or 2 level ACDF surgery patients **Significance/Strength of correlations:** fair to good correlations (0.57 to 0.71) between HUs and DXA, poor positive correlation (r = 0.26–0.28) between HUs and DXA with total subsidence **Limitations:** short follow-up period	C3–C6 or 3 surgical index vertebrae	High-subsidence patients (≥ 4.5 mm) had lower HU values (284.1 vs. 316.0) and T-scores (−0.5 vs. 0.1) than the low-subsidence patients (<4.5 mm). In comparison to their matched control group, patients with excess plate shift on one side had lower HUs (260.4 vs. 312.4) and higher total subsidence HUs and DXA positively correlated (r = 0.57–0.71) in the 1-level ACDF group, and (r = 0.59–0.66) in the 2-level ACDF group HUs (middle vertebra) were confirmed to be statistically associated with total subsidence after multivariate analysis
Pu et al., 2024 [14].	**Study design:** retrospective cohort study, single center **Objective:** to determine the effect of cervical HUs and T-scores on Zero-P subsidence after ACDF **Sample size**: *n* = 76; 12 months of follow-up **CT scanner:** UNITED, uCT510; 120 kV **Patient demographics**: ethnicity was not specified; the study was performed in China **Inclusion/exclusion criteria**: inclusion: cervical spondylosis patients undergoing single-level zero-p fusion, pre-op CT and X-ray within 1 week, exclusion: tumor, trauma, infection, bone disease, corticosteroid use, and multilevel fusions **Significance/Strength of correlations**: axial HUs and HUs of midsaggital, coronal, and axial had high odds ratios (OR = 0.925 and OR = 0.892) as independent risk factors for Zero-P subsidence **Limitations**: proposed future prospective studies, and longer follow-up studies	C2–C7	Significant differences were found between subsidence and non-subsidence groups in age, axial HUs, and midsaggital, midcoronal, and midaxial HUs. Lower cervical HU (<333.3 axial HUs, and <326.8 midsaggital, midcoronal, and midaxial HUs) was found to result in a higher risk of subsidence following zero-P fusion for cervical spondylosis
Abudouaini et al., 2023 [15].	**Study design:** retrospective, single center **Objective:** to determine if HUs correlate with titanium mesh cage (TMC) subsidence after ACCF **Sample size**: *n* = 64; minimum 12-month follow-up **CT scanner:** not specified **Patient demographics:** mean age: 51.29 years, 37 male, 27 female, ethnicities not specified **Inclusion/exclusion criteria**: inclusion: single to two-level ACCF patients using titanium mesh cage; exclusion: ACDF patients, endplate injury, revision surgery, trauma, C1–C7 tumor, severe osteoporosis, lack of radiological and clinical data, lost to follow-up **Significance/Strength of correlations:** fair correlations found between lower HUs and titanium mesh cage subsidence (r = −0.494) **Limitations**: relatively small sample size, proposed future follow-up prospective studies	Vertebrae above and below the artificial disk	Fair correlation found between lower HUs (HUs < 330.5) and titanium mesh cage subsidence after ACCF
Abudouaini et al., 2021 [16].	**Study design:** retrospective, single center **Objective:** to examine the effect of different hounsfield units on the clinical and imaging outcomes after cervical disk replacement **Sample size:** *n* = 127; follow-up duration: mean 17.67 ± 6.65 months **CT scanner:** not specified **Patient demographics:** mean age: 41.18 ± 8.57 years; 48 men and 79 women; ethnicities were not specified (study was performed in China) **Inclusion/exclusion criteria:** inclusion: 1- and 2-level CDR for cervical degenerative disk disease (C3–C7), radiculopathy/myelopathy patients refractory to conservative treatment for >3 months; exclusion: prior cervical instrumentation, trauma, tumors, severe osteoporosis, cervical infections, ankylosing spondylitis, rheumatoid arthritis, pregnant patients, neuromuscular disease patients, and patients with metal allergies **Significance/Strength of correlations:** significant differences between patients with HUs < 320 and HUs > 347 in subsidence (*p* = 0.011) and adjacent level degeneration occurrence (*p* = 0.032) **Limitations:** proposed future prospective and multicenter studies with larger sample size	Vertebrae above and below the artificial disk	Patients with lower HUs (<320) had significantly increased rates of implant subsidence, adjacent segment generation, and loss of intervertebral space height, but these were not associated with worse clinical outcomes
Ji et al., 2020 [17].	**Study design:** retrospective, single center **Objective:** to determine the risk factors for titanium mesh cage (TMC) subsidence following ACCF **Sample size:** *n* = 73; 24-month follow-up **CT scanner:** not specified **Patient demographics:** mean age: 58.18 years; 37 males and 36 females; ethnicities were not specified; the study was performed in China **Inclusion/exclusion criteria:** exclusion: tumor, trauma, posterior fusion, infection, and prior spinal instrumentation **Significance/Strength of correlations:** significant differences found between the ratio of anterior endplate, alignment of titanium mesh cage, and global cervical HU value (*p* < 0.001, *p* = 0.002, and *p* < 0.011) **Limitations:** possible lack of applicability to general population due to presumably predominantly Chinese patient population	C3–C6	Ratio of anterior endplate, alignment of titanium mesh cage, and global cervical HU value (<333) were significantly associated with subsidence
Wang et al., 2022 [18].	**Study design**: retrospective review, single center **Objective:** to examine the effect of cervical HUs on early titanium mesh cage (TMC) subsidence after ACCF **Sample size:** *n* = 85; pre-op CT within 1 week before surgery, at least 12-month follow-up **CT scanner**: PHILIPS, Brilliance; tube voltage 120 kV **Patient demographics**: mean age: 59.01 ± 7.97; ethnicities were not specified; the study was performed in China **Inclusion/exclusion criteria**: inclusion: cervical spondylosis patients, and ACCF by same team of surgeons; exclusion: spine infection, tumor, trauma, metabolic bone disease, endplate damage, hormone usage, and incomplete radiologic data **Significance/Strength of correlations**: HUs of the upper vertebral body and endplate were significantly lower in the subsidence than the non-subsidence group (*p* < 0.0001) **Limitations**: possible lack of applicability to general population due to presumably predominantly Chinese patient population	Upper and lower vertebral body and endplate of operative level	Lower pre-op CT HU (<275) of the inferior vertebral body is an independent risk factor for early titanium mesh cage subsidence after ACCF
Mei et al., 2024 [19].	**Study design**: retrospective review, single center **Objective:** to examine risk factors for early subsidence of 3D-printed artificial vertebral body (3D-PAVB) after ACCF surgery **Sample size:** *n* = 66; mean follow-up time 11.68 ± 1.55 months **CT scanner**: Siemens, Germany; voltage 120 kV **Patient demographics**: 36 males and 30 females; ethnicities not specified; the study was performed in China **Inclusion/exclusion criteria**: inclusion: cervical and spondylosis diagnosis; exclusion: spinal infection, trauma, tumor, metabolic bone disease, endplate injury, and adjacent vertebral disease **Significance/Strength of correlations**: patients with subsidence had a significantly lower average HU (*p* < 0.01) **Limitations:** small sample size; only examined risk factors for early subsidence due to short follow-up time	Upper and lower vertebral body of operative level	Independent risk factors for 3D-PAVB subsidence include smoking and lower HUs of the lower vertebrae (<272)

## Data Availability

No new data were created or analyzed in this study. Data sharing is not applicable for this article.
